# Supplementary material for: Evidence Supporting the Regulatory Relationships through a Paracrine Pathway between the Sternum and Pectoral Muscles in Ducks
Source: Genes (Basel). 2021 Mar 24;12(4):463. doi: 10.3390/genes12040463 (PMC8063953; doi:10.3390/genes12040463)
Supplement: Supplementary file 1 [file genes-12-00463-s001.pdf]

Evidence supporting the regulatory relationships through a paracrine pathway between the sternum and pectoral muscles in ducks

Yanying Li [1], Hehe Liu [1,*], Lei Wang [1], Yang Xi [1], Jiwen Wang [1], Rongping Zhang [1], Liang Li [1], Lili Bai [1] and Ahsan Mustafa [2]

Table S1. Primers used in Real-time PCR.

Gene	Sequence(5′-3′)	Length (bp)	Tm (°C)	Accession
DKK1	F: 5′ GCCAACCTCAGCAACTTCAACC 3′<br>R: 5′ GCTTGCAGATCTTGGACCAG3′	90	60	XM_027460718.1
OGN	F: 5′ CCACTGCCAAAGGAAACAGC3′<br>R: 5′ TGCAAAGGCTCCGTCTTCAA 3	147	60	XM_005025623.4
NOG	F: 5′ CTATTTCCCTGCCCGAGGAC 3′<br>R: 5′ AGCCCGTCGTAAAACTCCAG 3′	124	60	XM_005012255.3
GDF5	F: 5′CAGAGGCGGAAGAGAAGAGC3′<br>R: 5′TCCAGTCATCCCAGCCCATA3′	127	60	XM_021271325.2
SPP1	F: 5′ACAGACTTTCCCACAGACGC3′<br>R: 5′TCGATTTCACCATGCTGGCT3′	130	60	XM_005012779.4
BGLAP	F: 5′AGGAATTACGTCTATGACAGCAG3′<br>R: 5′GAAGCGCCGGTAAGCCTC3′	144	60	XM_027444528.1
CTSK	F: 5′GTGGCACGGAGGCTGATTT3′<br>R: 5′ATGGACACCCAGCGTATGC3′	78	54	XM_021277116.2
CXCL12	F: 5′GGAGGAGAAACCCGTCAG3′<br>R: 5′CTTGGGATCAATGCACACTT3′	169	54	XM_027459880.1
KCNA1	F: 5′AGCCCAGTTTCCCAATAC3′<br>R: 5′CCTCAGAGAACATGTCCAAG3′	182	54	XM_027449919.1
DCN	F: 5′GGATTTGGGCCAGTGTG3′<br>R: 5′TTGTATCAGGGGGAAGGTC3′	106	54	XM_005012380.4
MB	F: 5′CATCTGGGGGAAAGTGGAG3′<br>R: 5′GCTGGGTGAGGACGGTAAC3′	179	56	XM_005015343.4
LGI1	F: 5′TGGTTAGTGGAGTGGCTGG3′<br>R: 5′GCGCTTCTTATATTCTGGTGG3′	81	56	XM_027460399.1
β-actin	F: 5′ GCTATGTCGCCCTGGATTTC 3′<br>R: 5′ CACAGGACTCCATACCCAAGAA 3′	168	60	EF667345.1
GAPDH	F: 5′ AAGGCTGAGAATGGGAAAC 3′<br>R: 5′ TTCAGGGACTTGTCATACTTC 3′	254	60	AY436595.1

Table S2. Quality control of sequencing data.

Samples			Read Number [a]	Gbase Number [b]	GC Conten[c]	%≥ Q30[d]
Muscle	Un-calcified group	1	26,577,425	7.95	55.4	94.27
		2	38,501,610	11.51	54.04	94.12
		3	20,848,355	6.23	54.46	94.63
	Calcified group	1	21,122,560	6.32	55.26	94.71
		2	28,005,284	8.37	54.75	94.25
		3	20,517,575	6.13	54.35	94.38
Bone	Un-calcified group	1	26,400,703	7.89	55.95	92.62
		2	26,876,524	8.04	54.78	92.11
		3	31,243,733	9.33	54.86	94.00
	Calcified group	1	20,380,914	6.09	53.47	94.23
		2	25,080,949	7.50	56.00	92.39
		3	31,456,118	9.40	52.27	93.65

Note: a, the all number of pair-end reads in clean data; b, all number of bases in clean data; c, GC content of clean data; d, the percentage of quality value of clean data which not less than 30 bases.

Table S3. Differentially expressed genes in sternum between the calcified and un-calcified groups.

GENEID	Un-calcified group			Calcified group			Log2FC	P-Value
	1	2	3	1	2	3		
NRIP3	7.951851	6.609469	18.23261	0.008237	0.048368	0.120105	-7.5359	0.041494
OXTR	9.338109	8.296275	9.819655	0.283126	0.168611	0.513294	-4.8303	4.37E-05
LOC106018734	0.029199	0.04716	0.044285	0	0	0.005425	-4.47499	0.002796
DCT	3.470349	2.258641	2.80462	0.043289	0.006707	0.372938	-4.33465	0.001851
NDP	21.42148	12.78028	23.26256	0	0.114948	2.78731	-4.3074	0.023309
LOC106014639	1.439508	0.97347	1.423693	0.031253	0.01006	0.153908	-4.29667	0.00159
SCUBE2	36.4866	20.67186	45.73994	0.647147	0.21496	5.585666	-3.9963	0.04189
CPAMD8	434.9546	320.6637	642.7726	5.908142	1.200813	81.23045	-3.9846	0.035813
LEPR	1.220496	0.952486	0.792419	0.039783	0.018185	0.129606	-3.9827	0.002022
B3GALT2	0.727956	0.805849	0.538566	0.021959	0	0.121111	-3.85649	0.001839
LOC101801254	3.629584	2.698413	1.262	0.339812	0.076093	0.110707	-3.84929	0.027413
GDF5	18.95313	9.697635	23.2812	1.055629	0.214626	2.829878	-3.66288	0.017423
MAB21L1	3.423135	1.737226	2.05918	0.048907	0.243812	0.348186	-3.49372	0.013875
CSMD2	0.441999	0.460858	0.764974	0	0.005326	0.1534	-3.39336	0.012324
TUBAL3	1.705461	1.139188	1.142938	0.019502	0.255421	0.114275	-3.35694	0.003905
RASD1	39.91399	31.14056	17.72804	2.976563	3.014392	2.907212	-3.3187	0.014521
PTN	16.07891	12.01935	11.10621	1.167804	0.311849	2.660652	-3.2432	0.00775
OSTN	1.381611	1.858661	2.116875	0.142446	0.10769	0.315964	-3.24234	0.002074
CALCR	0.661914	0.946032	0.787635	0.05116	0	0.20866	-3.20479	0.002335
VIT	217.1154	183.8863	293.1541	17.40116	6.358801	54.83952	-3.1427	0.012554
LOC106018005	0.48041	0.247394	0.400006	0.045337	0.041002	0.043524	-3.11846	0.008237
HOXC9	14.07427	16.32704	19.31729	1.031055	0.492362	4.50307	-3.0444	0.002044
HMGCLL1	0.802809	0.904438	0.463263	0.06771	0.123306	0.073655	-3.0358	0.039327
KCNA6	9.495874	6.916637	5.698896	0.419527	0.058974	2.226487	-3.0311	0.012795
LRP1B	0.805773	0.535701	1.222395	0.027742	0.008819	0.277469	-3.0293	0.047562
LOC101794397	140.2543	79.89693	202.3566	15.68322	18.14294	20.12578	-2.96923	0.025519
ZDHHC1	8.364427	9.013741	10.91057	0.86533	0.622128	2.127956	-2.968	0.001815
TNMD	110.5825	118.3161	63.71753	14.57431	5.163548	18.03155	-2.95372	0.008289
LOC106015160	4.159529	4.216012	4.801508	0.355114	0.278752	1.07835	-2.94409	0.000307
SLC5A11	1.56542	1.257561	2.358745	0.045025	0.024517	0.61746	-2.915	0.025326
HOXB9	19.42359	16.18418	20.37839	0.911803	1.06911	5.568727	-2.8906	0.001429
SMOC2	249.3469	175.2611	265.5212	16.88778	7.839869	70.83028	-2.8524	0.005963
LOC106020081	16.17008	15.56104	16.29049	1.202415	0.547274	5.047755	-2.8206	0.008915
BMP3	10.54406	11.49545	12.12175	0.560502	0.466045	3.894972	-2.7952	0.006335
AFF3	8.907452	8.766712	13.07477	0.53685	0.335105	3.584891	-2.7864	0.009346
LOC106019837	0.571517	0.517736	0.709377	0.127399	0.041093	0.097451	-2.75771	0.001214
LOC101798535	28.24625	19.13359	31.58975	3.549784	4.198695	4.30593	-2.71174	0.003929

RNF43	2.60732	1.988372	2.85553	0.414218	0.185289	0.559824	-2.68418	0.001698
MEOX1	1.677799	0.865361	1.028807	0.209927	0.057211	0.292929	-2.673	0.047344
GRIN1	0.101602	0.166837	0.134468	0.011956	0	0.051467	-2.6674	0.010634
LOC101798873	0.598482	0.521548	0.412814	0.025936	0.087021	0.1347	-2.6298	0.002355
B3GAT1	3.106032	2.425443	4.048993	0.185894	0.274269	1.100385	-2.618	0.013202
CHADL	30.70835	24.48937	48.06767	3.142977	0.505209	13.1734	-2.618	0.0353
ADAMTSL1	28.05537	20.29667	24.9468	3.306829	0.71216	7.951614	-2.6143	0.002754
GATA5	9.573972	10.96695	10.49194	0.678955	0.563415	3.863703	-2.6035	0.00837
KCNH5	0.256001	0.405922	0.336511	0.050372	0.09139	0.02422	-2.5886	0.012304
NALCN	0.241092	0.258615	0.142973	0.026083	0.019987	0.060782	-2.5885	0.027525
BCAS1	1.267159	1.17309	1.586774	0.355787	0.047977	0.267031	-2.58577	0.001957
PTHLH	4.402006	5.892469	5.532465	0.411058	0.127788	2.124545	-2.571	0.006198
IGSF10	32.67925	16.26839	27.5596	4.754591	1.651751	6.749505	-2.5399	0.039261
KCNA1	29.05998	15.92337	30.05541	0.575084	0.021855	12.5452	-2.5134	0.028484
CHRFAM7A	1.555198	1.482119	1.196245	0.325753	0.053684	0.367359	-2.5031	0.001459
IL23R	21.59926	41.85236	50.72437	1.869553	0.603368	17.93921	-2.4838	0.046843
BOC	32.51343	24.85108	36.22298	3.797395	4.921857	8.452562	-2.44627	0.002172
LOC101803712	0.491548	0.442759	0.613166	0.076007	0	0.208928	-2.44121	0.006069
FIBIN	670.1268	552.5574	1099.658	85.1788	128.2427	218.8531	-2.42556	0.021074
THBS1	948.3018	1123.133	809.7905	170.9016	35.8663	373.3391	-2.3123	0.00464
COL21A1	1.606243	1.817302	1.691717	0.175391	0.528357	0.425534	-2.1794	0.000396
MMP27	15.7778	14.53811	16.27657	1.819079	1.72994	6.976183	-2.1462	0.014221
PRELP	776.186	756.475	1029.651	123.6073	136.5921	339.978	-2.09399	0.00435
LOC101789438	153.2853	98.70581	103.8945	27.95269	27.30802	31.06804	-2.0435	0.006723
NOV	304.4508	419.9108	293.6447	72.57359	88.25279	101.5117	-1.95625	0.003645
SLC16A14	0.85808	0.649603	0.579204	0.109327	0.126213	0.303382	-1.9532	0.007757
TDRP	6.241984	8.042384	3.414557	1.194889	1.733319	1.668561	-1.94497	0.032366
TSPAN6	141.6958	97.9724	205.4355	32.25227	39.92972	46.085	-1.91209	0.025738
RBP4	1.574506	1.526812	1.802339	0.240994	0.542779	0.526899	-1.90355	0.000766
NR4A2	11.74806	8.206923	6.237819	2.63866	1.603261	2.781651	-1.89889	0.018109
ZNF521	27.52182	37.99744	40.52342	13.20537	8.916916	7.167447	-1.85618	0.004232
TPH1	1.005403	0.647075	0.54306	0.220342	0.309903	0.078216	-1.85134	0.027189
HOXA10	1.173747	0.81228	0.601546	0.239476	0.168812	0.312551	-1.84385	0.022468
CHRD	6.408659	5.347376	4.02893	1.417596	1.443155	1.817789	-1.75442	0.006145
LOC101803042	15.67314	9.255095	15.89676	3.094493	4.47956	5.171745	-1.67943	0.014393
DCN	1957.486	1538.674	2163.053	498.2934	553.7261	793.8094	-1.61633	0.003433
LOC106016770	0.812297	0.958241	1.060375	0.347542	0.281747	0.30715	-1.59601	0.001061
ABCA12	0.178626	0.117677	0.142421	0.05943	0.039914	0.052487	-1.53085	0.00678
XKR5	4.975388	3.707576	5.644324	1.793853	2.000024	1.259011	-1.50359	0.007113
ST6GALNAC1	0.22474	0.264436	0.260571	0.120557	0.070093	0.078089	-1.4802	0.001344
LOC101797717	1.720915	1.31375	1.030962	0.627256	0.384012	0.499781	-1.42793	0.015962
KIF25	1.682629	1.991479	1.55385	0.672065	0.836112	0.435546	-1.42742	0.003281

LOC106015433	0.186174	0.134585	0.129425	0.058849	0.059217	0.065591	-1.2935	0.00823
LOC101792065	3.034459	2.441122	3.357726	1.216002	1.394142	1.014101	-1.28527	0.003912
SYNPO	34.02654	33.12393	42.79539	12.88858	16.39658	16.37677	-1.26773	0.002893
BHLHE40	65.52251	68.03352	76.02857	33.96953	23.75835	29.4006	-1.26632	0.000706
LOC101801758	25.72944	23.69924	19.34187	11.01015	9.360557	8.394791	-1.25745	0.002795
LOC106019138	3.052811	4.585155	3.180596	1.56332	1.53035	1.452162	-1.25089	0.013158
ITGA11	19.13985	18.82838	15.68348	9.169636	6.890468	6.862071	-1.22688	0.001586
COL6A1	247.6848	271.2957	295.787	141.5378	89.69759	123.4164	-1.2	0.001787
PHC3	3.868747	2.831444	3.249042	1.742367	1.072966	1.916442	-1.0722	0.011777
C1QTNF5	16.40511	16.57356	13.49428	8.553001	6.568149	7.042322	-1.06821	0.002245
IFRD1	27.69373	33.49925	24.93785	53.13395	67.11099	67.28996	1.122557	0.003155
MPC2	37.10359	35.05405	41.92141	74.51873	105.6059	69.12337	1.127548	0.017525
RAN	55.59413	61.92789	59.38528	123.6971	137.6021	126.3735	1.131846	0.000111
COL4A4	0.321465	0.404155	0.543991	0.696751	0.903322	1.224327	1.153558	0.035882
P2RY1	3.52696	2.699935	3.995487	8.098862	6.969931	7.687647	1.154543	0.001141
C2CD2	4.02793	4.973728	4.643376	11.67915	9.324483	9.862683	1.177657	0.001682
LOC101790525	3.547384	3.701888	3.704992	8.031364	6.380428	10.50944	1.185883	0.017895
CDC42SE1	36.74679	51.09073	45.59354	91.99565	120.0104	92.48406	1.190301	0.004948
COMTD1	2.324873	2.96087	2.130499	6.140439	4.823345	6.038346	1.196955	0.002894
SERINC2	7.857722	8.347746	11.88128	21.1581	25.27141	18.06617	1.199313	0.007649
SUCLG2	6.436686	5.64741	9.352528	16.97616	15.47025	17.54623	1.221638	0.001776
KIF15	2.900966	2.067845	2.644462	6.777313	5.053244	6.170563	1.241498	0.003518
MCM4	11.58667	11.66104	12.57807	28.34999	34.37457	22.77975	1.254999	0.007908
KPNA2	13.95036	16.51505	13.49465	37.68046	34.38338	33.36924	1.262063	0.000217
RBBP4	40.90131	37.89401	41.77938	96.20062	117.9568	75.14466	1.26265	0.010576
ARPC2	77.99091	84.7671	79.60006	191.895	211.7166	181.6951	1.272053	0.000226
FAM49B	6.251708	7.43434	5.800058	15.94474	17.15986	14.47646	1.287942	0.000516
CTNNB1	140.3147	145.9034	152.4572	378.2742	418.9876	274.0967	1.2882	0.038613
VWA2	0.796997	1.639351	0.857736	2.838482	3.371004	2.082355	1.331815	0.022621
RAPGEF4	0.346708	0.391686	0.685072	1.265082	1.019601	1.324848	1.342403	0.006719
SEC14L1	10.94615	15.40634	14.33588	36.69235	37.27708	29.58205	1.34766	0.00174
LOC101802919	0.88602	1.098434	1.252644	2.497848	3.534453	2.225154	1.350996	0.015419
MYO9B	9.959741	6.787769	10.69758	28.00483	25.46514	18.39665	1.3888	0.02277
LOC101789621	20.49502	19.5467	19.88099	61.20515	56.94009	39.02187	1.391125	0.008868
ATP5A1	92.0416	85.24198	93.6592	253.8799	282.8708	177.9258	1.399302	0.009232
AHCY	22.02347	23.77137	19.68752	61.2123	67.86225	44.99684	1.410501	0.006291
DPH6	0.187302	0.141028	0.18493	0.515247	0.406992	0.454043	1.423014	0.001164
STON2	1.007012	0.99097	1.385243	3.137412	2.951851	3.085091	1.439207	0.000161
LOC101799686	5.267997	4.427953	5.653047	15.45131	9.752176	16.44729	1.440199	0.014355
PPL	1.023212	0.601155	0.721191	2.285843	2.028519	2.187416	1.470903	0.000693
LY86	19.57925	20.24074	15.23719	56.7488	47.15736	49.57468	1.479056	0.000561
IFIH1	1.434686	1.437861	1.902599	4.151401	5.624157	3.601042	1.486095	0.010048

ITGB5	13.6367	29.91911	13.19291	70.61391	42.75143	45.94948	1.4892	0.039031
SLC47A1	0.490357	0.662156	0.811447	1.865974	2.061715	1.591147	1.4906	0.003189
FKBP3	24.91068	19.26107	21.91394	52.35004	79.15164	55.67885	1.502021	0.009316
SERPIND1	15.11637	11.62795	11.33922	32.43629	46.99712	28.92366	1.508554	0.01443
LOC106020586	47.67565	48.74715	45.49001	102.2362	114.9738	186.8909	1.509711	0.029539
LOC106020294	5.754336	6.645367	5.603067	17.42371	24.13627	12.47115	1.585572	0.024088
HIPK2	14.70562	16.21467	16.07765	57.738	44.30883	39.59303	1.591558	0.004447
BMP4	3.969753	6.52819	2.839728	12.43688	15.78744	12.16983	1.5986	0.004884
BMP4	3.969753	6.52819	2.839728	12.43688	15.78744	12.16983	1.5986	0.004884
FAM49A	4.470098	5.61869	5.293669	17.3768	19.19311	10.09828	1.601153	0.020389
SMAD6	11.35871	17.13143	11.83088	48.5123	42.76287	31.80284	1.61	0.019679
SLC13A3	0.166934	0.25344	0.286175	0.438114	0.940179	0.788137	1.616458	0.03341
LOC101801508	0.051184	0.088929	0.030043	0.181435	0.143359	0.207189	1.644522	0.008865
ANO1	1.625075	2.23242	1.302892	6.829411	4.757377	4.941253	1.679365	0.006121
LOC101795891	2.990576	1.771505	3.371803	10.38873	7.224318	8.803513	1.699426	0.004132
TRAIP	2.664135	2.179917	2.246542	8.909659	9.432947	4.829388	1.708404	0.021506
ETS1	7.587851	11.0957	6.823845	33.94615	30.34244	19.19636	1.710601	0.013988
COLEC12	27.80652	35.15668	29.55545	124.9773	121.2324	61.79307	1.735127	0.02514
SH3BGRL3	47.25931	59.01602	43.02083	175.339	221.575	100.2434	1.735526	0.031371
LMNB1	3.526051	2.213124	3.818856	12.4246	12.03619	7.851587	1.757301	0.007989
PRCP	7.124947	11.56694	9.781454	34.57241	38.80655	22.98157	1.758831	0.009915
CCT7	34.73263	34.66828	28.91555	110.0504	147.1765	76.13477	1.761584	0.019066
CD2AP	8.958364	9.500479	7.964974	35.21754	34.26743	20.68601	1.770824	0.010767
EHD4	3.012339	4.02124	3.995752	14.05299	13.74271	10.03879	1.778357	0.002567
WNT7B	0.005144	0.008211	0.002999	0.018395	0.023966	0.014195	1.79	0.024106
KCNJ2	1.73599	3.146359	0.824841	8.504628	6.628418	4.609947	1.7905	0.03262
SLC9A7	3.654194	4.978975	1.969085	14.73221	12.75866	9.234752	1.7924	0.016554
JARID2	1.141593	1.984982	1.981156	7.532977	4.308818	5.853542	1.792615	0.012494
PPFIBP2	2.527133	2.386466	1.848043	9.799917	9.109651	4.539752	1.794101	0.028723
NUAK1	5.155195	7.064415	3.635565	23.91648	18.44408	12.67045	1.795291	0.018358
PTP4A3	3.756481	5.044618	3.313319	16.27397	14.55758	11.96704	1.820838	0.001651
FABP3	16.7957	12.6689	10.64245	38.70795	34.01399	70.79952	1.839339	0.042126
NABP1	3.425955	3.464481	3.945134	13.66446	16.66874	8.641751	1.846772	0.016187
LY6D	1.595568	1.697966	1.574187	3.79335	5.285585	8.581253	1.859182	0.03941
TMPPE	0.879796	1.005799	0.976144	3.771195	4.778129	1.953957	1.875876	0.036965
HEXB	38.5986	57.28873	36.74797	155.228	187.2236	147.0502	1.883849	0.001024
PLXND1	6.317325	11.42379	9.08983	38.73988	39.48471	21.19852	1.8897	0.048817
ACOT7	3.601351	4.555705	2.812216	14.70643	15.86493	10.38442	1.900599	0.004572
ZEB2	6.763697	5.856699	7.325591	31.76923	27.89774	15.04396	1.905221	0.02279
LOC106014545	19.73838	34.87162	21.72962	109.1378	98.71095	79.68656	1.913235	0.002022
LOC101804245	0.910188	1.645073	1.133033	2.603556	5.037804	6.360729	1.924617	0.037468
LOC101796117	0.565096	0.4951	0.510015	1.866531	2.72444	1.390756	1.929604	0.019729

PNPLA2	5.896679	6.304268	4.608757	11.74904	23.87786	28.70128	1.936157	0.035357
SH3BGRL	13.75917	16.86825	15.11441	77.87353	58.0105	41.75765	1.957384	0.013756
LOC101802187	10.08598	7.126559	7.488008	42.95153	28.43662	25.66778	1.974273	0.011392
UHRF1	3.325341	3.744038	3.46164	16.44046	16.77588	8.926381	2.000639	0.014763
LOC106017261	2.002702	1.703867	1.360448	10.08518	5.324183	5.089701	2.016349	0.034835
IL18R1	0.093017	0.259803	0.177998	0.734538	0.836601	0.590823	2.026052	0.003206
HDAC9	1.337406	0.569694	1.420887	5.473826	3.749429	4.458336	2.039514	0.003732
RASGRP3	0.183948	0.186885	0.130637	0.766687	0.883325	0.464988	2.076422	0.012955
BAZ1A	3.234913	5.820325	3.953379	17.19889	12.63449	25.80564	2.096629	0.022579
ST3GAL6	4.742782	3.967755	3.187174	21.35825	13.87569	16.01134	2.106735	0.004458
LOC101798226	0.930596	2.993634	0.904976	9.073153	7.646304	4.381325	2.1274	0.040968
HMGA2	0.016576	0.076002	0	0.188856	0.116117	0.10103	2.1327	0.043966
LOC101798089	1.210104	1.83154	1.611587	7.157115	8.144705	5.126279	2.1343	0.023589
PIK3R5	0.275117	0.736966	0.435403	2.130139	2.275264	1.977254	2.1406	0.001144
DYRK3	2.712633	3.126472	3.04818	15.63992	14.85127	8.725606	2.1417	0.043263
PPARG	1.223211	2.15292	1.148304	6.929165	8.684961	4.460879	2.149591	0.014963
LAPTM5	8.357541	5.836697	6.44989	31.73641	38.45115	22.18781	2.1618	0.033988
MYO1B	10.56211	16.62081	15.00332	74.63937	73.92979	40.58426	2.164712	0.012599
LOC101805210	0.272421	0.312589	0.097635	0.78986	0.938873	1.342335	2.1695	0.027407
CIDEA	0.361642	0.89846	0.831556	2.916307	3.474926	3.030284	2.1713	0.000524
LOC101795069	0.141944	1.053413	0.323917	2.703204	2.005409	2.143133	2.1731	0.008493
LOC101799867	0.043135	0.04182	0.063117	0.258383	0.270777	0.141163	2.1786	0.048483
PLVAP	34.92379	46.26038	34.86683	160.4332	258.4655	107.5654	2.181576	0.036811
PTTG1	4.739076	6.325451	2.982966	23.38322	25.06165	15.40972	2.184478	0.006069
DOK4	2.601989	3.964431	6.264418	23.13502	20.70559	15.12818	2.2003	0.011906
LOC101789604	10.08081	3.385919	9.422885	36.27121	44.58895	24.79022	2.2065	0.028845
LOC101789696	1.342586	2.006281	1.221129	6.838214	9.261161	5.032071	2.2091	0.041331
HEY1	9.78934	24.99361	11.06894	67.53808	93.28262	53.61972	2.225524	0.011151
LOC101791711	0.752716	1.117924	1.357482	6.417245	4.745023	3.989496	2.230719	0.005762
PLAU	1.308516	3.868452	3.723186	13.92142	15.78027	12.17679	2.2343	0.001439
ZFAND2A	1.385571	4.82577	3.084347	15.06513	16.13177	12.91301	2.246469	0.001071
RHOC	7.460227	20.86202	9.050102	66.8876	54.0882	59.79723	2.2741	0.00113
LOC101799532	2.889374	3.115865	4.090796	17.49376	20.63626	11.06096	2.2846	0.041672
GPR18	0.012373	0.048646	0.202796	0.346736	0.493803	0.449413	2.289719	0.009333
ALDH1A2	3.767517	8.04281	4.334321	34.11784	20.44645	25.05452	2.302053	0.007504
SPRY4	1.042434	2.220295	0.899786	9.450127	6.31383	5.206336	2.33282	0.013837
PCSK6	3.735176	5.190556	4.449743	32.13299	20.01204	17.79381	2.386504	0.013561
LOC101798211	0.323334	0.556475	0.309067	2.401105	1.76869	2.079775	2.394159	0.00107
TNFRSF9	0	0.159907	0.108346	0.50972	0.551341	0.349782	2.3949	0.009361
USP35	0.333664	0.312495	0.353234	2.280452	1.441637	1.573206	2.405587	0.005355
MME	10.4352	11.25166	9.903596	61.38771	79.09336	27.99347	2.41497	0.038228
LOC101800964	0.228674	0.168571	0.30565	1.709271	1.023598	1.033506	2.421796	0.011398

KCTD20	7.665673	9.518785	6.611509	52.61045	23.51782	51.38292	2.421835	0.022262
LOC101796634	0.013645	0.022467	0.054781	0.189472	0.08044	0.230119	2.459776	0.04243
SH2B2	0.244254	0.110257	0.060919	0.641409	0.708222	0.975684	2.484749	0.005351
P2RY13	0.462731	0.244666	0.329888	2.381753	2.038915	1.403819	2.489319	0.005549
IL1R2	0.139719	0.23135	0.174362	0.963633	1.445076	0.686014	2.504342	0.019026
LOC101798440	0.153946	0.832991	1.241815	3.306338	3.6872	6.001366	2.543638	0.016291
SIAH2	1.726231	2.580808	2.428777	17.31539	6.46949	16.40717	2.576985	0.032865
FKBP1B	0.159204	0.275346	0.164826	1.244795	1.398786	0.9782	2.595166	0.001431
PLBD1	2.314336	3.08758	1.776346	13.58631	20.17005	10.31125	2.618012	0.013634
NEURL1	0.50904	0.702141	0.576902	3.539159	4.411221	3.03264	2.6188	0.015371
BTLA	0.618728	0.397982	0.907034	4.850765	2.418182	4.711234	2.6387	0.046922
LOC101805205	1.489936	2.825849	1.834873	13.56572	17.25405	7.696222	2.646645	0.018505
ENPP2	3.557621	8.133619	5.309019	36.00423	42.04696	29.71576	2.664286	0.00135
PRKAR2B	0.850647	1.518892	0.749686	7.528024	7.126542	5.293984	2.677024	0.001532
LOC101797844	0.323133	0.654265	0.037638	2.761503	2.371019	1.378441	2.6813	0.031812
LOC106015692	0.105752	0.541404	0.194947	2.494321	1.236279	1.689813	2.6863	0.040859
ATP6V1D	19.9666	25.98322	17.94123	147.2525	192.9221	71.64248	2.688318	0.030736
KCNJ4	0.00119	0.00117	0.028641	0.068729	0.076087	0.055179	2.6896	0.009883
ITGA8	1.572013	1.76827	1.359361	14.49932	9.020735	7.213367	2.709185	0.016735
TNFAIP3	1.81933	2.102012	1.14458	14.15188	13.74723	5.422637	2.717567	0.030087
FBP1	0.449675	0.682079	0.225942	3.128737	3.65996	2.226232	2.731156	0.004345
LOC106018047	0.332188	0.305966	0.257469	2.418651	2.507112	1.131062	2.757598	0.018082
BASP1	6.079831	9.637861	5.032116	50.04017	72.20691	21.97468	2.797119	0.047884
MYO1A	0.095709	0.320299	0.055933	0.929323	1.505662	0.845675	2.7973	0.032237
SLCO4C1	0	0	0.100911	0.267991	0.271303	0.173942	2.8213	0.011747
ADAMTS7	5.792074	7.071779	4.677126	55.01281	52.20503	19.03568	2.847522	0.035149
LOC101795211	1.639196	1.072016	0.972799	10.90994	10.77433	5.108766	2.862509	0.016047
GLI1	0.31413	1.585399	0.500906	8.304923	3.928045	5.39544	2.8765	0.048328
SLC8A3	0.778385	24.96998	1.073496	67.98414	59.27873	73.1201	2.9013	0.007932
MLXIPL	0.01744	0.034448	0.002474	0.087349	0.168819	0.161524	2.9418	0.031806
KNDC1	0.013577	0.017783	0.031627	0.144839	0.116705	0.222695	2.942593	0.012009
ITGB2	1.919172	1.513892	1.387433	12.43995	18.6747	6.532937	2.965303	0.035586
FAM78A	1.619749	1.734455	1.155129	14.56984	15.90041	5.123501	2.980638	0.038021
LRRC17	3.65342	3.114853	5.067158	39.41214	36.26461	21.6103	3.039135	0.00668
DLX5	3.375723	15.59523	3.15886	71.95979	64.7884	45.71145	3.0435	0.00889
P2RX5	0.915786	0.766477	0.670812	6.6386	7.098517	5.678494	3.0446	0.004411
GPRIN3	0.090274	0.104178	0.076371	0.783905	1.163821	0.330712	3.072623	0.049948
CTSC	56.25732	71.17295	56.81176	673.7112	627.1908	249.4492	3.072921	0.027595
ARHGAP25	0.779238	1.071745	0.695716	8.78321	10.72313	2.873724	3.135514	0.04894
LOC101798404	0.529967	0.649878	0.770194	7.661583	6.826137	3.084349	3.171709	0.020914
LOC101791902	0.709701	0.827335	0.607216	9.636782	4.950529	4.809803	3.177296	0.022297
SYK	0.360157	0.697247	0.463531	5.482999	6.116791	2.273467	3.189276	0.026078

LOC101794528	46.48601	127.3229	69.93727	706.3287	938.7271	578.4133	3.189361	0.003651
LOC101796470	0.010837	0.148484	0.180087	1.017786	1.47392	0.676819	3.2227	0.049033
PLTP	5.191178	7.592242	5.936633	63.18446	82.10884	29.90818	3.226359	0.026905
HRG	0	0.01433	0.033889	0.127937	0.189192	0.13842	3.2399	0.007786
LOC101800757	0.329837	0.318518	0.175617	3.889388	2.677542	1.225859	3.241473	0.039519
SSTR2	0.098551	0.289948	0.286849	1.897616	3.172169	1.450125	3.271149	0.019975
SASH3	0.760374	0.572386	0.190139	6.024977	6.835986	1.938747	3.280677	0.044003
LOC101804611	2.061893	5.711555	2.546879	42.06315	39.15617	20.04299	3.294537	0.012349
LOC106015547	0.109816	0	0	0.341723	0.456902	0.296638	3.3181	0.006579
LOC101797023	0.048673	0.071873	0.048463	0.562951	0.636856	0.514274	3.342263	0.000146
LOC106018505	5.731707	14.44875	6.51639	107.644	78.88863	85.27366	3.347838	0.00087
CCDC67	0.021348	0.042967	0.025346	0.396774	0.210614	0.316007	3.364395	0.006897
TSPAN8	0.24398	0.807421	0.546723	4.342897	4.846722	7.332216	3.3699	0.029646
LOC106015766	0	0	0.01877	0.069748	0.07246	0.052087	3.3717	0.002823
INHBE	0	0.072889	0.011901	0.342326	0.190272	0.346682	3.3744	0.02201
DSCAML1	0	0.034168	0.003611	0.120051	0.177743	0.098326	3.390281	0.010153
ATP8B3	0.029991	0.029778	0	0.263651	0.234701	0.140267	3.4175	0.028527
STEAP3	3.630008	4.011876	1.738139	36.98146	47.65452	16.39716	3.429093	0.029358
CGN	0.074187	0.089373	0.047731	0.931286	0.95917	0.517811	3.5107	0.035112
TNFSF13B	0.408826	0	0.841846	6.492781	4.58831	3.201761	3.5135	0.038051
SLC4A8	0.113205	0.135826	0.14094	2.096468	1.79531	0.884216	3.614362	0.015985
RBM47	0.518962	0.467668	0.423312	7.317682	7.456293	2.617253	3.624652	0.028596
TNN	0.220144	0.241802	0.150514	3.489889	1.693143	2.713545	3.68854	0.009587
LOC101789532	2.901834	6.25316	2.945159	70.45073	39.5309	47.53926	3.702446	0.00654
LOC101805370	0	0.291799	0	1.32064	1.535265	0.970871	3.7131	0.006788
LOC101800022	0.925994	2.217834	0.981735	18.91198	23.84617	11.7626	3.724142	0.00894
AADAC	0.314146	1.290676	0.666834	7.019416	15.93996	7.238913	3.732651	0.034382
LRRC7	0.055534	0.102573	0.07539	1.205673	1.419412	0.517532	3.750491	0.023598
AGPAT1	0.003028	0.01876	0.007615	0.128279	0.156192	0.112091	3.7535	0.005713
MFNG	0.081741	0.200991	0.068911	1.824152	1.880478	1.067767	3.7625	0.027632
LOC101794862	0	0.032071	0.050844	0.49623	0.26192	0.400321	3.804445	0.006743
EPHX4	0.026173	0.111496	0.070619	1.25737	0.706542	0.946658	3.8047	0.027557
LOC101797638	0.019854	0	0	0.088646	0.079885	0.110103	3.81087	0.001505
LOC106018256	1.722168	2.76726	1.948964	39.34869	29.88308	22.21626	3.82818	0.00466
LOXHD1	0	0.002907	0.009749	0.062279	0.039243	0.08404	3.874	0.041217
TP53I11	7.391105	23.10123	10.52349	260.5485	153.2427	200.8621	3.90552	0.003682
SLCO2B1	2.381345	6.577194	2.032758	72.91413	60.40844	32.15789	3.912227	0.013253
PLS1	0.458255	0.205305	0.165197	4.552285	5.528671	2.60505	3.936145	0.010235
STMN3	0.091961	0	0	0.412654	0.668045	0.347881	3.9574	0.035228
TFEC	0.263892	0.172604	0.195071	4.390154	3.220611	2.321204	3.975072	0.00665
CYTH4	0.347102	0.529538	0.338787	8.835799	7.914888	2.807843	4.008263	0.031093
LRP4	0.97497	1.377861	0.759369	23.45923	20.94032	7.050863	4.047176	0.034306

LOC106015870	0.347474	0.725699	0.429502	9.800171	12.05684	2.996945	4.047871	0.046113
SLA	0.424271	0.508292	0.418959	5.468375	9.506321	7.66825	4.066405	0.003702
DRD4	0.013117	0.10383	0.040878	0.970742	0.928793	0.755278	4.072213	0.000306
CD200	0.024584	0.012082	0	0.238624	0.245808	0.134357	4.0769	0.028886
SPI1	2.44378	2.388783	1.717064	40.11237	55.7487	15.33612	4.085564	0.041377
PTPN22	0.772491	0.993749	0.816118	19.35353	20.84215	5.361801	4.140927	0.043899
RGS1	1.309703	0.952047	0.498878	22.53485	17.96005	9.396445	4.175721	0.015192
PRKCH	0.472254	0.461685	0.576956	11.15295	10.97417	5.374581	4.186	0.044704
LOC106014796	0.027156	0.007245	0.021539	0.372501	0.366129	0.290081	4.2008	0.004826
TFPI	0.250188	1.662321	0.200547	11.57522	21.60359	7.51338	4.267349	0.037983
BCL2L15	0.098415	0.128026	0	1.72471	1.767772	0.90168	4.2784	0.036628
LOC101792090	0.011509	0.101783	0.037288	0.89525	1.332381	0.794401	4.3269	0.025941
DNAJC12	0.310983	0.53537	0.54255	7.208862	11.67242	10.14155	4.38517	0.002166
CITED4	1.368469	2.14762	0.474708	30.55164	42.21389	11.01171	4.391809	0.043344
APCDD1	0.612358	0.932	0.390377	23.07507	16.37704	6.134711	4.558409	0.041867
SPATA13	0.152429	0.236618	0.137476	5.578064	5.017758	2.114617	4.593373	0.019399
DKK1	0.362625	0.403531	0.095493	8.034694	10.84617	2.9617	4.663898	0.038827
CIDEC	0.377776	0.89525	0.537912	17.57758	23.40843	11.43049	4.855212	0.008201
SUSD3	0.01244	0.005888	0.006716	0.258389	0.153357	0.370263	4.964648	0.015774
CNRIP1	0.422203	0.577934	0.227772	17.47838	17.05653	4.576299	4.993307	0.040621
LOC106014932	0.065001	0.486039	0.025153	6.153954	7.112709	5.2417	5.005481	0.000436
LOC106015741	0.052382	0	0.02331	1.06076	0.573745	0.850673	5.037065	0.004796
SLC15A2	0.03915	0.168992	0.093574	3.498038	4.450673	2.03824	5.048781	0.010066
GALNT9	0.07259	0.090523	0.065662	3.405182	3.608373	1.01422	5.132999	0.035492
DCSTAMP	0.120229	0.415396	0.223068	13.9114	9.469485	3.844993	5.165318	0.038868
PRDM1	0.052316	0.239341	0.159215	8.791799	4.759729	3.119098	5.208447	0.032704
IFI30	3.200633	2.627677	3.622243	150.9655	162.1336	36.98793	5.211171	0.046882
LEF1	0.162668	1.033892	0.234489	22.72486	26.67642	6.141035	5.278442	0.045727
PANX3	0.497319	6.92295	0.372212	106.8344	155.4403	71.99778	5.422798	0.010992
RPS6KA2	0.024975	0.031849	0.054242	1.666969	1.983638	1.181704	5.443224	0.00252
NT5E	1.196303	0.930595	0.912582	82.48615	22.34332	46.02132	5.633154	0.047989
ATP6V0D2	1.404587	3.231416	1.304909	107.0444	146.3454	44.33732	5.647162	0.030685
NXPH2	0.096247	0.270907	0	2.726093	6.850923	9.937963	5.732053	0.037915
SAMD5	0.007127	0	0	0.166534	0.110555	0.110967	5.7668	0.019403
LOXL4	0.811774	7.448712	2.177733	267.7148	150.2189	169.7046	5.815	0.033554
HNMT	0.119477	0.141029	0.055431	6.1923	11.51412	4.121417	6.110389	0.031184
LOC101797729	0.053052	0	0	0.998589	1.764763	0.916893	6.1163	0.045894
MYL3	0.041248	0.306173	0.424966	19.65388	18.3864	28.01377	6.4182	0.018606

Table S4. The enriched pathways of modules.

Enriched pathways	p-value	Nodes
Focal adhesion	9.42E-12	COL6A6, COL6A2, COL9A3, COL4A4, VEGFA, IGF1, SPP1, ITGB3, COL4A3
ECM-receptor interaction	2.85E-11	COL6A6, COL6A2, COL9A3, COL4A4, SPP1, ITGB3, COL4A3
TGF-beta signaling pathway	5.65E-06	BMP7, BMP6, NOG, DCN
Cytokine-cytokine receptor interaction	1.05E-04	CXCL12, BMP7, TNFRSF11B, VEGFA

Table S5. List of cluster analysis between proteins secreted by sternum and DEGs in the pectoral muscle.

Differential gene in the pectoral muscle	Secreting protein in the sternum
EGLN3	DCN, GDF5
MB	CTSK, CXCL12, VASH1, DKK1, ITGB3, BMP7
WASF1	CHGA
PTK2B	ASPN

Table S6. List of cluster analysis between proteins secreted by pectoral muscle and DEGs in the sternum.

Differential gene in the sternum	Secreting protein in the pectoral muscle
GABRA1	HYAL2
COL4A4, UMODL1	CHST13
COL10A1, GNG4	MRPL32
ITGB6, PIL5	HTATIP2, PLA2G12B
GRIN2A	LRRTM4, GABRG2
OSTN, COL9A3, DCN, KCNA1	LGI1

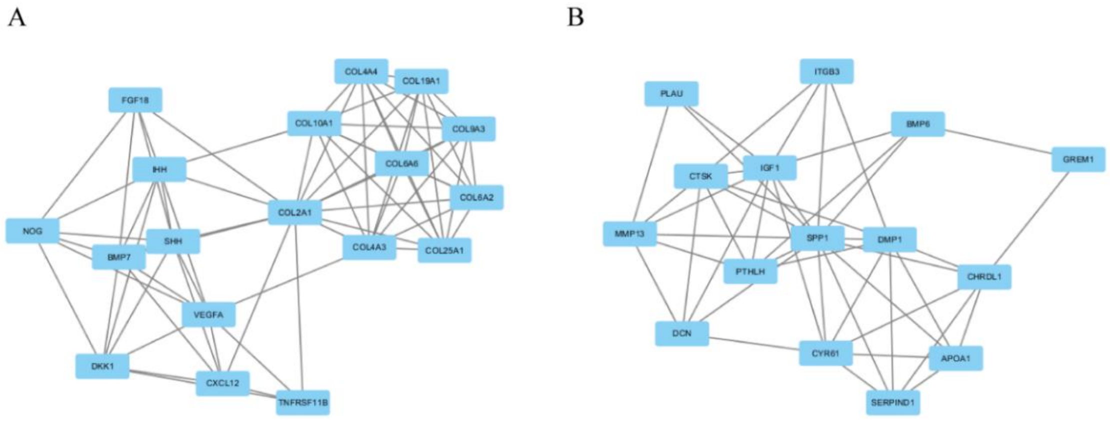

Figure S1. Top two modules from the PPI network in the calcified and un-calcified sternum. The squares represent the DEGs in modules, and the lines show the potential interactive relations between the DEGs. A: module 1. B: module 2.

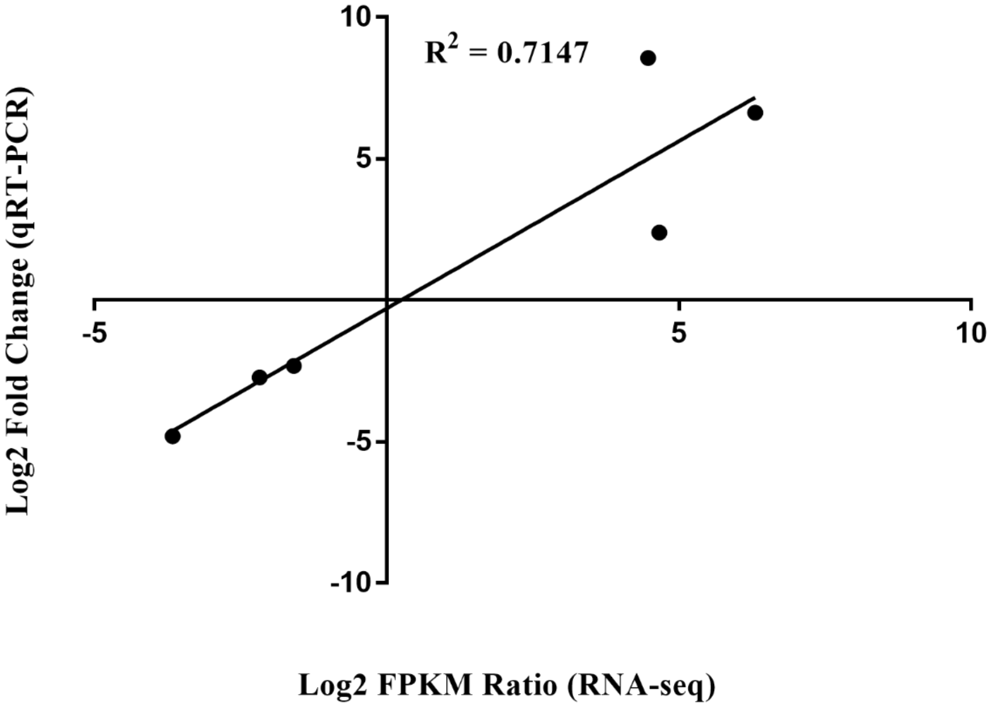

Figure S2. Validation of RNA-seq results by qRT-PCR. Correlation plots indicating the relationship between qRT-PCR results (fold change; Y-axis) of 6 selected genes and the corresponding data from RNA-seq analysis (X-axis).

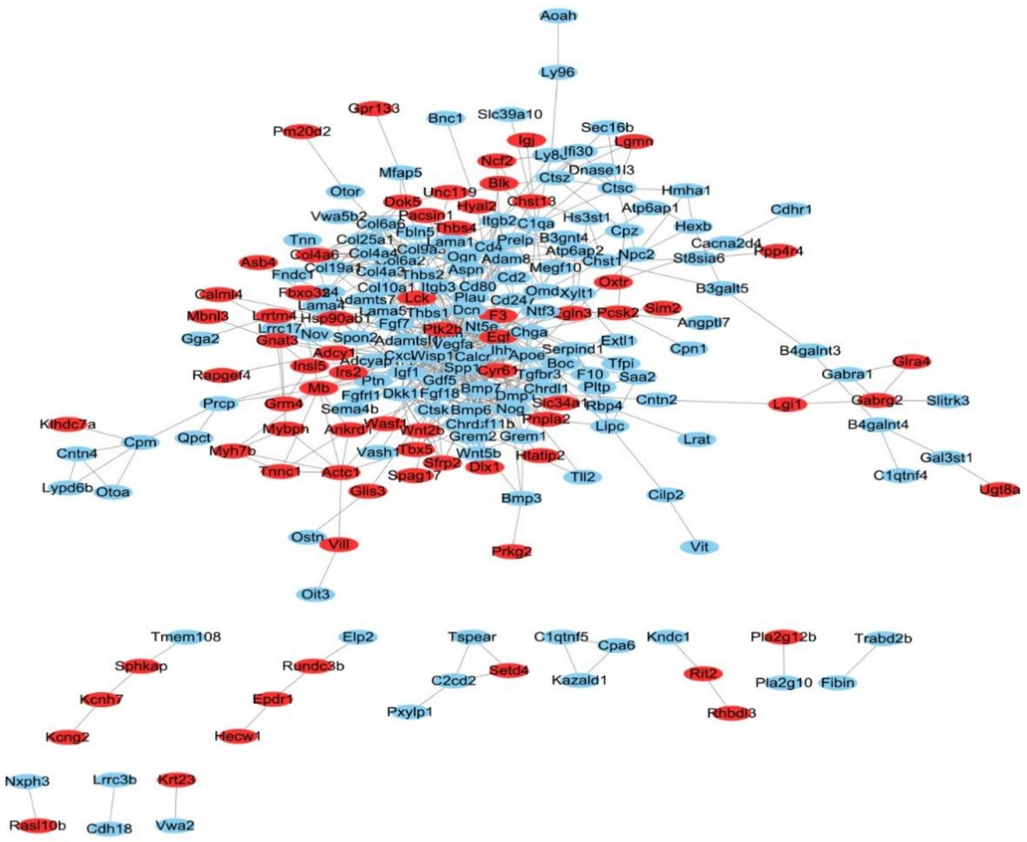

Figure S3. The PPI networks of DEGs, whereby red nodes represent DEGs in pectoral muscle and blue nodes represent proteins secreted by bone.

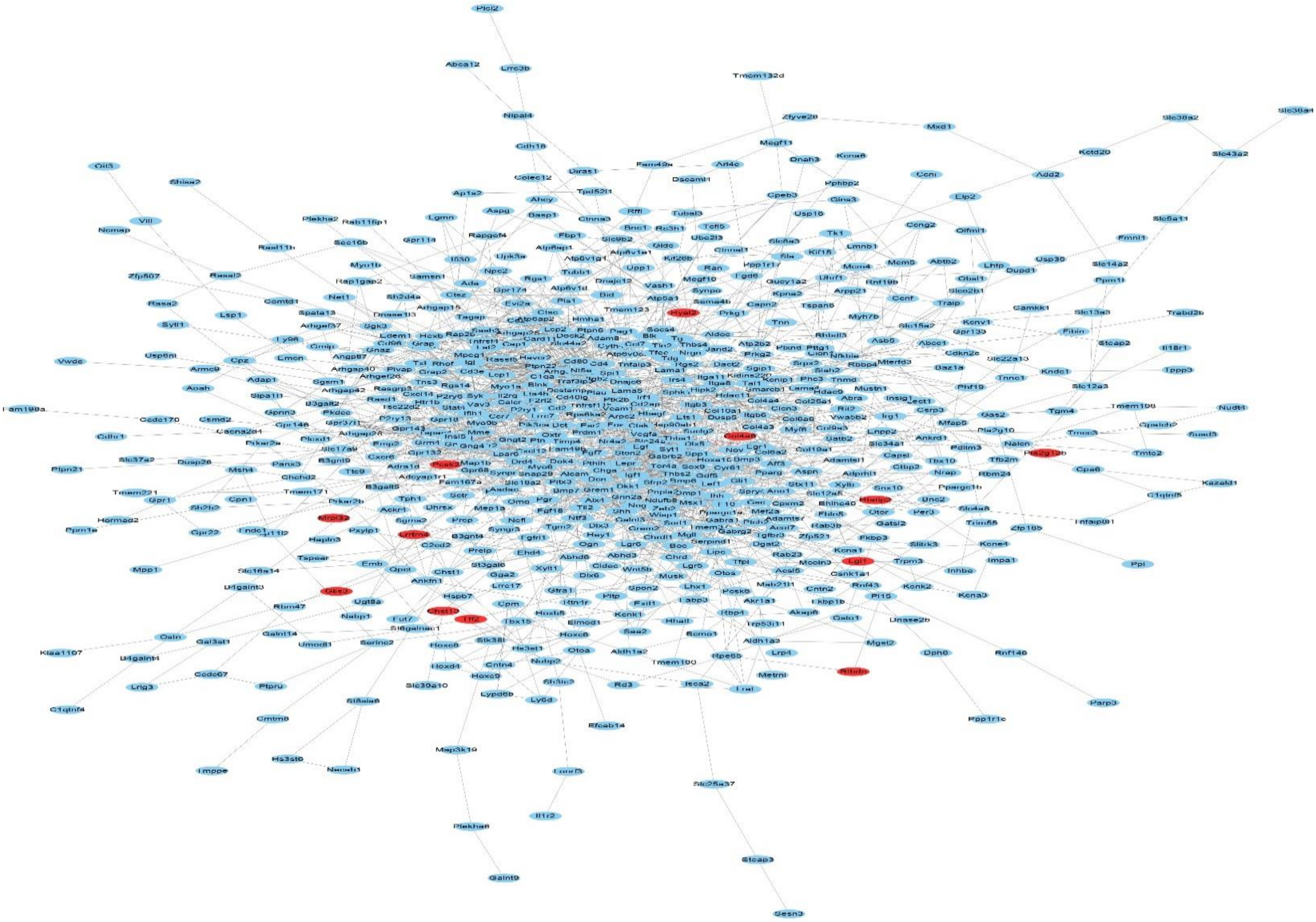

Figure S4. The PPI networks of DEGs, whereby red nodes represent proteins secreted by pectoral muscle and blue nodes represent DEGs in bone.
